# A Rare Case of Aleukemic B‐Cell Acute Lymphoblastic Leukemia Presenting as *Rapidly Progressive* Severe Low Back Pain in an Adolescent With a History of Anxiety and Depression

**DOI:** 10.1155/crpe/6527741

**Published:** 2026-11-04

**Authors:** TingTing Huang, Lingbin Meng, Linghua Zheng, Xue Han, Ephraim Ansa-Addo, Feng Hong

**Affiliations:** ^1^ Pediatrics PrimaryOne Health Columbus Ohio USA; ^2^ Division of Medical Oncology The Ohio State University College of Medicine Columbus Ohio USA osu.edu; ^3^ Division of Microbiology Infection and Immunology The Ohio State University College of Medicine Columbus Ohio USA osu.edu

## Abstract

**Introduction:**

Musculoskeletal complaints are a frequent cause of pediatric emergency visits. While adolescent back pain is typically benign, it can occasionally signal underlying malignancy. Distinguishing organic pathology from functional disorders is particularly challenging in patients with pre‐existing psychiatric comorbidities, leading to potential diagnostic delays.

**Case Presentation:**

We report a 17‐year‐old female with a history of anxiety and depression presenting with two weeks of severe lumbar pain and progressive gait difficulty. Initial evaluations, including spinal radiography and complete blood count, were unremarkable. Her symptoms were initially attributed to musculoskeletal strain. However, as symptoms persisted, her psychiatric history may have contributed to diagnostic overshadowing when psychogenic factors were subsequently considered. Rapid functional deterioration prompted further workup, revealing markedly elevated inflammatory markers and lactate dehydrogenase (LDH). Magnetic resonance imaging (MRI) demonstrated diffuse marrow signal abnormalities. Subsequent bone marrow biopsy confirmed B‐cell acute lymphoblastic leukemia (B‐ALL). Cytogenetics analysis showed a normal karyotype.

**Discussion:**

ALL rarely initially presents with isolated back pain in adolescents, commonly resulting in delayed diagnosis. This delay can be further exacerbated by diagnostic overshadowing, a cognitive bias in which a patient’s psychiatric history leads clinicians to prematurely attribute severe somatic symptoms to functional or psychogenic etiologies. This case also highlights that early leukemic bone marrow infiltration may occur despite normal peripheral blood counts and unrevealing plain radiographs. Therefore, persistent symptoms and rapid clinical deterioration despite initially reassuring investigations should prompt reconsideration of the diagnosis and additional evaluation, including selected laboratory studies, such as LDH and inflammatory markers, and advanced imaging (MRI) when clinically indicated.

**Conclusion:**

This case underscores the importance of maintaining a broad differential diagnosis and looking beyond psychiatric comorbidities when evaluating adolescents with persistent or nocturnal back pain accompanied by rapid functional decline, despite initial reassuring radiographs and hematologic studies. In such patients, additional laboratory assessment and MRI may be considered when the clinical course is disproportionate to an expected musculoskeletal injury, helping to identify serious underlying pathology and reduce the risk of diagnostic delay.

## 1. Introduction

Acute lymphoblastic leukemia (ALL) is the most common pediatric malignancy, accounting for approximately 25% of childhood cancer diagnoses [1]. Although systemic findings such as fever, lymphadenopathy, and hepatosplenomegaly typically prompt hematologic evaluation, musculoskeletal pain is the presenting symptom in up to one‐third of cases [2, 3]. This pain results from rapid expansion of the medullary cavity by malignant lymphoblasts, causing increased intraosseous pressure and periosteal stretching [4]. However, isolated low back pain as the predominant initial manifestation of ALL is rare and has been described primarily in case reports and small case series [5].

Diagnosis is particularly challenging in “aleukemic” presentations, characterized by normal or low peripheral white blood cell counts without circulating blasts [6]. In such cases, peripheral blood studies fail to reflect true tumor burden, frequently leading to misdiagnosis as juvenile idiopathic arthritis, osteomyelitis, or mechanical strain. These delays may persist for weeks and increase the risk of morbidity [7, 8]. Diagnostic overshadowing further complicates evaluation in patients with psychiatric comorbidities when physical symptoms are prematurely attributed to anxiety or depression, resulting in incomplete investigation [9].

This report describes an aleukemic presentation of B‐ALL in an adolescent whose predominant presenting symptom was rapidly progressive low back pain. The case highlights the limitations of relying on normal initial radiography and peripheral blood counts to exclude serious pathology and illustrates how a pre‐existing psychiatric diagnosis may contribute to premature attribution of severe physical symptoms to psychogenic causes. We also discuss the role of additional laboratory evaluation, particularly lactate dehydrogenase (LDH) and inflammatory markers, in distinguishing occult malignancy from benign musculoskeletal or psychosomatic conditions [10, 11]. Magnetic resonance imaging (MRI) may be considered when persistent or nocturnal back pain is accompanied by rapid functional deterioration despite initially reassuring investigations.

## 2. Case Report

### 2.1. History of Present Illness

A 17‐year‐old female with a medical history of anxiety and depression presented to an outpatient clinic with a two‐week history of lower back pain. The pain was localized to the lower lumbar spine, described as sporadically severe (reaching up to 10/10 in intensity), and notably worsened at night. It intermittently radiated to the bilateral lower extremities and was accompanied by numbness and difficulty ambulating. She also exhibited recent behavioral changes, characterized by increased anxiety, depression, and irritability.

A review of systems was positive for generalized fatigue but was otherwise negative for constitutional symptoms; she reported no fever, night sweats, changes in appetite, or weight loss. She also explicitly denied recent trauma, strenuous activity, bruising, bleeding, or recurrent respiratory or gastrointestinal infections. She reported no changes in bowel or urinary habits and no saddle anesthesia. Her medications prior to presentation were limited to acetaminophen and ibuprofen taken as needed for pain control; she was not taking any psychiatric medications or systemic corticosteroids. Her family history was non‐contributory.

### 2.2. Prior Evaluations

The patient had two prior emergency department (ED) visits within the preceding 2 weeks. During the first visit, physical and neurologic examinations were unremarkable; lumbar radiographs and urinalysis were normal. She was discharged with a diagnosis of musculoskeletal strain and offered ibuprofen. Four days later, she visited a different ED with persistent back pain, nausea, and vomiting. Repeat evaluations, including viral panels and pregnancy testing, were negative, and she was again discharged with symptomatic management.

### 2.3. Outpatient Follow‐Up

At an outpatient clinic follow‐up the next day, the patient’s condition had deteriorated significantly. She required a wheelchair for mobility due to severe pain and subjective weakness secondary to fatigue. Despite this rapid functional decline, she remained hemodynamically stable. A comprehensive physical examination was largely benign. Notably, there was an absence of pallor, petechiae, lymphadenopathy, hepatosplenomegaly, and focal spinal or bone tenderness.

Despite her reliance on a wheelchair, a detailed objective neurological examination was unremarkable. She demonstrated full, symmetric motor strength of 5/5 on all extremities, intact sensation globally, and normal deep tendon reflexes. When ambulation was assessed, her underlying gait was normal, but she could only walk briefly due to the pain. Gait pattern was normal despite limited tolerance. Mobility was restricted primarily by avoidance and fear, and bilateral straight leg raise tests were negative. Given the discordance between the severity of her reported pain and the benign physical findings, especially in the context of a significant psychiatric history, a psychogenic etiology was initially considered. However, screening laboratory investigations were performed to rule out organic pathology.

### 2.4. Diagnostic Workup

Initial laboratory evaluation (Table 1) revealed a normal CBC without peripheral blasts or cytopenias. Metabolic testing was notable for mild transaminitis and a borderline low serum bicarbonate. However, inflammatory markers were markedly elevated, with an erythrocyte sedimentation rate (ESR) of 78 mm/h and a C‐reactive protein (CRP) of 14.7 mg/dL. Due to the discrepancy between the normal hematologic profile and the severe inflammatory response, an urgent referral was made to rheumatology for the evaluation of suspected juvenile idiopathic arthritis. The patient was seen within 5 days by rheumatology after an urgent referral was made.

**TABLE 1:** Laboratory investigations at outpatient clinic and hospital.

Laboratory parameter	Outpatient follow‐up	Hospital admission	Reference range	Units
White Blood Cell (WBC)	9.1 (Normal)	7.7 (Normal)	4.5–11.0	× 10^3^/μL
Hemoglobin (Hb)	14.5 (Normal)	12.7 (Normal)	12.0–16.0	g/dL
Platelet Count	172 (Normal)	123 (Low)	150–450	× 10^3^/μL
ESR	78 (High)	71 (High)	< 20	mm/h
CRP	14.7 (High)	8.6 (High)	< 1	mg/dL
LDH	—	7329 (High)	325–650	U/L
Uric Acid	—	6.4 (Normal)	2–7	mg/dL
AST	57 (High)	78 (High)	15–50	U/L
Serum Bicarbonate	18 (Low)	26 (Normal)	20–29	mEq/L

### 2.5. Hospital Course

Upon evaluation in the rheumatology clinic, the patient was immediately admitted to the hospital due to profound functional decline and acute behavioral changes, including marked worsening of anxiety and depression, new‐onset suicidal ideation, and increased irritability without evidence of psychosis. These symptoms represented a significant deterioration from her previously mild, well‐controlled anxiety and depression. A psychiatry consultation was obtained on admission, and supportive therapy with ongoing psychiatric management was recommended. Further diagnostic workup revealed persistently increased ESR and CRP. Most notably, LDH level was found to be severely elevated (7329 U/L), while uric acid remained within normal limits. A repeat CBC showed stable leukocyte and hemoglobin levels, but new‐onset borderline thrombocytopenia (123 × 10^3^/μL).

### 2.6. Imaging and Pathology

MRI of the spine was performed (Figure 1). T2‐weighted sequences demonstrated diffuse marrow signal abnormalities throughout the thoracic and lumbar spine and sacrum. Multifocal areas of increased signal intensity and enhancement were noted, suggestive of a marrow‐replacing process such as malignancy, chronic recurrent multifocal osteomyelitis, or multifocal infection. There was no evidence of discitis or compression of intraspinal contents.

**FIGURE 1:**
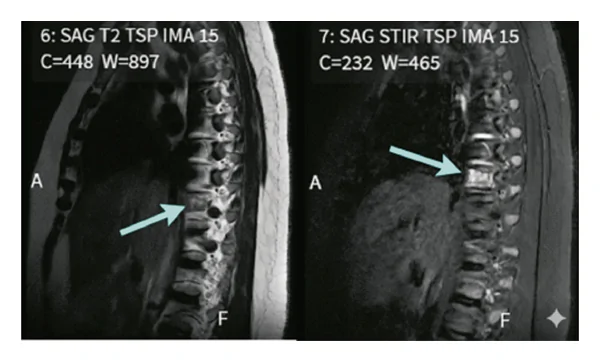
Magnetic resonance imaging (MRI) of the thoracic, lumbar, and sacral spine. STIR and T2‐weighted sequences demonstrate discrete areas of abnormal, increased signal intensity scattered throughout the thoracic and lumbar vertebral bodies, as well as the sacrum. These regions of abnormal marrow signal, which exhibit contrast enhancement, are indicated by the arrows.

Given the imaging findings and elevated LDH, a bone marrow aspiration and biopsy were performed. The biopsy confirmed the diagnosis of B‐ALL involving a hypercellular bone marrow (71.5% blasts). Cytogenetics analysis showed normal chromosomes (FISH analysis not performed).

### 2.7. Follow‐Up and Outcomes

The patient was subsequently transferred to the oncology service for induction chemotherapy. Following the initiation of leukemia treatment and adequate pain control, her clinical status improved dramatically. Her severe back pain completely resolved, allowing for a full recovery of her mobility and ambulation without the need for assistive devices. Furthermore, the treatment of her underlying malignancy and physical pain led to a significant improvement in her acute psychiatric symptoms. Her anxiety and depression returned to baseline, and she no longer experiences suicidal ideation or irritability.

## 3. Discussion

This case highlights the diagnostic complexity of an infiltrative bone marrow process presenting initially as mechanical back pain (Figure 2). The clinical course illustrates three critical pitfalls in pediatric medicine: the challenge of evaluating adolescent spinal pain, the risk of diagnostic overshadowing in patients with prior psychiatric diagnoses, and the limited sensitivity of the CBC in screening for hematologic malignancy.

**FIGURE 2:**
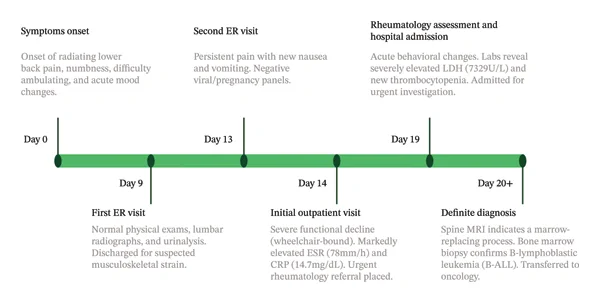
A structured chronological timeline showing the onset of symptoms, ED visits, outpatient visit, laboratory testing, rheumatology referral, admission, MRI, biopsy, and transfer to oncology, using the initial outpatient clinic visit as the anchor point (Day 0).

Although mechanical back pain is increasingly common in adolescents, pediatric spinal pain requires a high index of suspicion for organic disease. “Red flag” features should prompt urgent evaluation. In this patient, significant red flags were present, including nocturnal pain and rapid functional decline with loss of ambulation. Evaluating such presentations requires a balanced differential diagnosis. While mechanical musculoskeletal strain was initially suspected, the progression of nocturnal pain and mobility loss necessitated a broader evaluation. The differential appropriately expanded to include infectious processes (such as discitis or osteomyelitis), inflammatory bone disease (such as chronic recurrent multifocal osteomyelitis), and malignancy (including leukemia and Ewing sarcoma) [12].

Anxiety and depression can sometimes present as unexplained musculoskeletal pain and backaches [13]. When a patient has a known history of psychiatric conditions, clinicians may prematurely attribute new or worsening physical back pain to their mental health. This misattribution is known as diagnostic overshadowing [14]. In this case report, earlier diagnostic decisions were appropriately focused on musculoskeletal etiologies; however, the patient’s underlying anxiety and depression, combined with an absence of focal neurologic deficits and the onset of acute irritability, may have acted as a possible contributor to diagnostic delay when clinicians began to consider functional neurologic symptom disorder. However, attributing severe red‐flag symptoms to a psychosomatic etiology without comprehensive advanced imaging constitutes a significant diagnostic risk. In the setting of severe pain and physical decline, behavioral changes should be interpreted as possible manifestations of organic disease. This case underscores that functional and somatic symptom disorders must remain diagnoses of exclusion after a thorough medical evaluation.

A further barrier to early diagnosis was the initially normal CBC. Although leukemia typically presents with cytopenias or peripheral blasts, their absence does not exclude malignancy. “Aleukemic leukemia” can present with bone marrow infiltration despite normal peripheral blood findings [15], highlighting the limitation of relying solely on CBC screening in children with persistent bone pain.

Biochemical markers ultimately guided the appropriate escalation of care. Elevated ESR and CRP indicated a systemic inflammatory process, shifting the differential toward infectious, rheumatologic, or malignant etiologies [16]. While LDH is a nonspecific marker of cellular injury and turnover that can be elevated in a variety of conditions—including infection, inflammation, and hemolysis—studies have explored its utility in evaluating these complex presentations [17, 18]. In this patient, the markedly elevated LDH, in combination with clinical deterioration, evolving thrombocytopenia, and abnormal marrow imaging, significantly increased the clinical concern for malignancy [17].

Given the combination of red flags and biochemical abnormalities, an MRI was pursued. The imaging revealed diffuse marrow signal changes demonstrating an abnormal marrow process; however, these findings alone did not definitively distinguish a malignant infiltrative process from a multifocal infection or inflammatory bone disease, such as chronic recurrent multifocal osteomyelitis. Ultimately, a bone marrow examination was required to establish the definitive diagnosis. While MRI is highly sensitive for detecting marrow‐replacing lesions and is far more sensitive than plain radiographs, which often appear normal in early disease, bone marrow aspiration and biopsy remain necessary for the definite diagnosis of leukemia. Although this single case does not dictate a universal diagnostic pathway for all adolescent back pain, it supports the consideration of checking LDH levels and pursuing an MRI when severe functional decline and red flag symptoms are present.

## 4. Conclusion

This case emphasizes that normal CBC results do not exclude leukemia and that psychiatric history should not preclude thorough investigation. While this report does not advocate for broad routine screening, clinical features such as nocturnal back pain and significant functional decline warrant careful evaluation. In these targeted clinical scenarios, LDH testing and MRI should be considered as part of a comprehensive diagnostic approach to aid in the timely identification of serious underlying pathology.

**Nomenclature**
ALL: Acute lymphoblastic leukemia
ED: Emergency department
ESR: Erythrocyte sedimentation rate
CRP: C‐reactive protein
LDH: Lactate dehydrogenase
MRI: Magnetic resonance imaging
CBC: Complete blood count

## Author Contributions

TingTing Huang: patient care, concept, data collection, analysis, writing, and manuscript acceptance. Lingbin Meng: data collection, discussion, and manuscript acceptance. Linghua Zheng, Xue Han, and Ephraim Ansa‐Addo: writing–review and editing and manuscript acceptance. Feng Hong: Concept, writing–original draft, review, editing, and final acceptance.

## Funding

There are no sources of funding.

## Ethics Statement

Ethical approval is not required for case reports.

## Consent

Written informed consent was obtained from the patient’s legal guardian for publication of this case report and accompanying clinical information. A copy of the signed consent form is available for review by the editor‐in‐chief of this journal on request.

## Conflicts of Interest

The authors declare no conflicts of interest.

## Data Availability

Data sharing is not applicable to this article as no datasets were generated or analyzed during the current study.
